# Nitric oxide, lipid peroxidation products, and antioxidants in primary fibromyalgia and correlation with disease severity

**DOI:** 10.2478/jomb-2019-0033

**Published:** 2020-01-23

**Authors:** Varsha Shukla, Das Siddharth Kumar, Mahdi Abbas Ali, Shweta Agarwal, Sukhanshi Khandpur

**Affiliations:** 1 King George's Medical University, Department of Rheumatology, Lucknow, Uttar Pradesh, India; 2 Era's Lucknow Medical University, Lucknow, Uttar Pradesh, India; 3 Career Institute of Medical Sciences and Hospital, Department of Medicine, Lucknow, Uttar Pradesh, India; 4 University of Lucknow, Department of Statistics, Lucknow, Uttar Pradesh, India

**Keywords:** fibromyalgia, severity, antioxidants, nitric oxide, azot-monoksid, antioksidanti, težina bolesti, fibromijalgija

## Abstract

**Background:**

Fibromyalgia syndrome (FMS) is characterized by altered pain perception with chronic, widespread musculoskeletal pain. The relationship between nitric oxide, oxidative stress and the severity of FMS has not been studied. This study evaluated NO levels in plasma, LPO products and antioxidants in Red Cell lysate in patients of FMS and correlated it with disease severity.

**Methods:**

105 FMS patients who fulfilled 1990 ACR Criteria and 105 age- and sex-matched healthy controls were recruited over two years from 2013 to 2015. Antioxidative enzyme activity was assessed by the estimation of catalase, glutathione peroxidase (GPx) and glutathione reductase (GR) and superoxide dismutase (SOD). Nitric oxide in plasma, MDA marker of lipid per - oxidation (LPO) in the lysate was donen for estimating oxidative stress. FIQR was used to assess the severity of fibromyalgia.

**Results:**

The catalase, superoxide dismutase, glutathione reductase and glutathione peroxidase levels were significantly low in patients than controls (p<0.001). Plasma NO levels and LPO were also significantly high (p<0.05). NO and LPO levels showed a significant positive correlation with FIQR (r: 0.57, 0.8 and p: <0.001) whereas a negative correlation was observed between antioxidants (Cat, GR and GPx, but not SOD) and FIQR.

**Conclusions:**

Low antioxidants and raised LPO in RBC lysate in patients with FM together with high plasma NO correlated with the severity of FMS.

## Abstract (sr)

**Uvod:**

Fibromijalgijski sindrom (FMS) karakteriše promena percepcije bola sa hroničnim, raširenim mišićno-skeletnim bolom. Odnos između oksidativnog stresa i težine FMS nije ispitan. Ova studija je vršila procenu nivoa NO u plazmi, LPO proizvodima i antioksidantima u lizatu crvenih ćelija kod pacijenata sa FMS i utvrđivala odnos sa ozbiljnošću bolesti.

**Metode:**

Odabrano je 105 FMS pacijenata koji su ispunili kriterijume ACR-a za 1990. godinu i 105 zdravih kontrolnih subjekata koji su odgovarali po starosti i polu i to tokom perioda od dve godine, od 2013. do 2015. Izvršen je uvid u aktivnosti antioksidativnog enzima procenom katalaze, glutation peroksidaze (GPx) i glutation reduktaze (GR) i superoksid dismutaze (SOD). Urađen je azot-monoksid u plazmi, MDA marker lipidne peroksidacije (LPO) u lizatu za procenu oksidativnog stresa. Za procenu težine fibromijalgije korišćen je FIQR.

**Rezultati:**

Katalaza, superoksid dismutaza, nivo glutation reduktaze i glutation peroksidaze bili su značajno niži kod pacijenata nego kod kontrolne grupe (p < 0,001). Nivoi NO u plazmi i LPO takođe su bili značajno visoki (p < 0,05). Nivoi NO i LPO su pokazali značajnu pozitivnu korelaciju sa FIQR (r: 0,57; 0,8 i p < 0,001) dok je negativna korelacija uočena između antioksidanata (Cat, GR i GPx, ali ne i SOD) i FIQR.

**Zaključak:**

Niski antioksidansi i povišeni LPO u RBC lizatu kod pacijenata sa FM zajedno sa visokom plazmom NO koreliraju sa težinom FMS.

## Introduction

Fibromyalgia syndrome (FMS) is a com mon, chronic, rheumatic disorder characterized by widespread pain, diffuse tenderness of muscles, tendons and joints, debilitating fatigue, stiffness, depression, and many other symptoms. It is estimated that the prevalence of FM is approximately 3-5% of the world population [1] [2]. Its pathophysiology and etiology remain unclear; it seems to be the outcome of a complex interaction of multiple factors, such as genetic, neuroendocrine, immune, psychosocial, and environmental behavioural [3] [4]. Studies have proposed that nitric oxide (NO) may have an important role in the severity of FMS symptoms [5].

Nitric oxide is gaseous in nature and is produced by catalyses of L-arginine by dioxygenase. It has a great capability of diffusion between body tissues and performs several important biological functions such as vasodilatation, modulation of nociception, immune function, neurotransmission, and excitation-contraction coupling [6] [7]. NO a free radical signal-transuding agent, has been shown to undergo a sequence of reactions, leading to the generation of reactive nitrogen species (RNS) in a variety of cells [8].

Lipid peroxidation is a process in which oxidants such as free radicals or non radical's species reacts with carbon-carbon double bond containing lipids, especially polyunsaturated fatty acids (PUFA). Lipid peroxidation produces a wide variety of oxidation product. The primary product of lipid peroxidation is lipid hydroperoxides (LOOH). Whereas the secondary product of lipid peroxidation produces many aldehydes amongst them malondialdehyde (MDA), propanal, hexanal and 4-hydroxynonenal (4-HNE) are widely studied as markers of oxidative stress [9]. It is important to acknowledge that lipid peroxidation is associated with the score of fibromyalgia impact questionnaire (FIQ) [10].

Antioxidants help prevent or stop cell damage caused by oxidants. Catalase, superoxide dismutase and glutathione enzymes are known as powerful antioxidants produced by the body and used as a biomarker of antioxidant defence [11]. These antioxidants control the harmful oxidative effect of reactive oxygen species (ROS). When free radical production exceeds the antioxidant capacity of the body, it leads to oxidative stress [12]. This condition is responsible for the cellular damage or injury which has been implicated in over a hundred disorders, including fibromyalgia.

Recent studies have shown the involvement of NO in the initiation of lipid peroxidation, which may have a role in the severity of FMS [8].

This study aimed to analyse the levels of NO, LPO and antioxidants in the patients of FMS and controls and correlate it with the severity of FMS through FIQR.

## Materials and Methods

This is a case-control study in which blood samples were collected from 105 FMS patient fulfilling the 1990 American College of Rheumatology (ACR) criteria for the diagnosis of FM [1] and 105 healthy controls having apparently no disease accompanying the patients. Age of patients and controls age ranged from 18 to 60 years, respectively. The subjects were selected from amongst people attending the outpatient clinic in the Department of Rheumatology, King George's Medical University, Lucknow, U.P. Informed consent was obtained from all subjects. Exclusion criteria included the presence of any inflammatory autoimmune diseases, diabetes mellitus, renal disease, known heart disease; use of any kind of antioxidant. The study protocol was approved by the Institutional ethics committee.

In patients with fibromyalgia, Depression was measured by the Centre for Epidemiologic Studies Depression Scale (CES-D), the overall impact of fibromyalgia with the Fibromyalgia Impact Questionnaire-Revised (FIQR), and the number of tender points at 18 standard sites on the body by applying 4 kg pressure with the thumb on specific body points. Tender points were also scored as 0-4 points. (0 -no pain, no reaction, 1 - painful but no reaction, 2 - painful but minimal reaction, 3 painful with irritation, 4 - painful with excessive irritation) [2].

### Preparation of RBC lysate

Laboratory parameters included preparation of RBC lysate. For this purpose patient and control blood samples were centrifuged at 3000 rpm for 15 min, which separated supernatant which is plasma and was stored at -80 °C for further biochemical estimation and the pellet that is RBC was again used for lysate preparation. The pellet was then washed twice with 1 mL normal saline (0.9% NaCl) and centrifuged at 3000 rpm for 10 minutes, and the supernatant discarded (normal saline). Now chilled distilled water (equal volume to plasma) was added in the pellet and centrifuge at 5000 rpm for 20 minutes. The supernatant form was lysate and pellet was discarded. The lysate was then used for the biochemical estimation.

Estimation of NO was done using nitric oxide Assay Kit in plasma of patients and controls. RBC lysate MDA as a marker for lipid peroxidation was determined using the thiobarbituric acid (TBA) method Ohkawa et al. [13] and antioxidative enzyme activity in the RBC lysate of patients and controls were done by cpectrophotometric analysis for the estimation of catalase (Cat) through Aebi's method [14], superoxide dismutase (SOD) by Mc Cord and Fridovich [15], glutathione reductase (GR) was measured by method of Hazelton and Lang [16] and glutathione peroxidise (GPx) by the method of Pagila and Valentine [17]. The results of the antioxidative parameters were expressed as unit/min/mg protein whereas lipid peroxidation content in the sample was expressed as nmol of MDA/mg protein.

### Statistical Analysis

Statistical Package for Social Sciences (SPSS) software, version 20.0 for Windows, was used for all statistical analyses. Data were not normally distributed as tested by using the Shapiro-Wilk test at 5% level of significance; therefore, non-parametric tests were used. To test statistical differences between patients and controls, Mann-Whitney U test was used and Spearman correlation coefficient (r_s_) to establish a correlation between antioxidants, oxidative stress marker (LPO) and nitric oxide, was used. Karl Pearson's correlation coefficient (r) was performed to find the pattern of association between the antioxidants, nitric oxide, lipid peroxidation and FIQR total score. The Spearman correlation coefficient (r_s_) is based on the ranked values for each variable rather than the raw data. Therefore, we applied Karl Pearson's correlation coefficient(r).

## Results

The mean age of FMS patient was 38.9 ± 10.1 years (range: 18-60 years) and mean age of healthy controls was 36.9 ± 9.6 years (range: 18-60 years). As expected, there was a female preponderance since FM is common in females (80% female and 20% male).

### Nitric oxide, lipid peroxidation and role of antioxidative parameters

A statistically significant difference in antioxidants like catalase, SOD, GR and GPx was found among FM patients and controls. Current study result represents a decreased level of antioxidants in patients with FMS compared to those in control (Table 1). A significant elevation in NO level and lipid peroxidation was seen in the FM patient than in control (Table 1), furthermore strong positive linear relationship was observed between FIQR total score and nitric oxide and lipid peroxidation, a strong negative linear relationship was observed for FIQR total score with the antioxidative enzymes (cat, GPx and GR) and no linear relationship was observed between FIQR total score and SOD (Table 2). Our results also represent a significant negative correlation between antioxidative parameters, MDA (a marker of oxidative stress) and nitric oxide (Table 3).

**Table 1:** Antioxidative parameters and NO level among FMS cases and control group (Mann-Whitney U test) Case group, the participant with fibromyalgia syndrome; control group, participant without fibromyalgia syndrome; Cat, catalase enzyme; SOD, superoxide dismutase; GR, glutathione reductase; GPx, glutathione peroxidise; FIQR, fibromyalgia impact questionnaire-revised, p< 0.001*significance; MDA, malondialdehyde; NO, nitric oxide

Variables	Median (Min.-Max.)		P Value
	Cases	Controls	
Cat (kU/min/mg protein)	2.39 (1.05–7.90)	12.09 (6.91–18.74)	<0.001*
SOD (μmol/min/mg protein)	40.43 (30.13–41011.00)	60.73 (40.16–76.00)	<0.001*
GR (μmol/min/mg protein)	1.19 (0.18–3.65)	13.59 (10.00–16.87)	<0.001*
GPx (μmol/min/mg protein)	18.45 (6.31–62.37)	58.00 (30.82v–79.85)	<0.001*
NO (mol/gr)	62.59 (0.75–458.65)	13.00 (8.00–20.00)	<0.001*
MDA (mmol/mg protein)	32.37 (6.83–45.01)	11.56 (8.00–27.54)	<0.001*
FIQR Total	76.00 (45.00–98.70)	5.00 (1.00–16.00)	<0.001*

**Table 2:** Karl Pearson’s product moment correlation between antioxidative parameters and nitric oxide with FIQR *= significant. FIQR = Fibromyalgia Impact Questionnaire Revised. (r) = Karl Pearson’s correlation Coefficient

Variables	FIQR Total Score (r)
Cat (kU/min/mg protein)	-0.880*
SOD (μmol/min/mg protein)	0.065
GR (μmol/min/mg protein)	-0.943*
GPx (μmol/min/mg protein)	-0.79*
Nitric Oxide (mol/gr)	0.569*
MDA (mmol/mg protein)	0.79*

**Table 3:** Spearman rank correlation between antioxidants, oxidative stress marker (MDA) and nitric oxide Cat = catalase, SOD = superoxide dismutase, GR = glutathione reductase, GPx = glutathione peroxidase, MDA =malondialdehyde, * = significance values

Antioxidants	MDA (Oxidative Stress Marker) (mmol/mg protein)	Nitric Oxide
Cat (kU/min/mg protein)	-0.736*	-0.622*
SOD (μmol/min/mg	-0.644*	-0.598*
GR (μmol/min/mg protein)	-0.678*	-0.619*
GPx (μmol/min/mg protein)	-0.692*	-0.669*

## Discussion

The results of this study show that catalase, SOD and glutathione reductase and glutathione peroxidase levels were found to be lower; LPO and NO levels were elevated in the FMS patients than in controls. We also observed a positive linear association of NO and LPO with FIQR and negative linear association of FIQR with antioxidative enzymes and no linear relationship was observed between FIQR and SOD. Furthermore, in the current study, we also found that LPO and nitric oxide were negatively correlated with the antioxidants. These lead us to the main discussion point whether the increased level of NO in the plasma and lipid peroxidation in RBC lysate of patients with FMS and decrease in antioxidants in RBC lysate of patients may contribute to pathogenesis or the severity of fibromyalgia. There exists a counter prevailing view that changes in NO level, lipid peroxidation and antioxidants in an individual may be due to fibromyalgia. Though these findings do not provide evidence of a pathogenetic relationship, the positive linear relationship of NO and LPO with FIQR and negative rela-tionship with antioxidants suggest that they may be related to the severity of FMS.

These findings are similar to the results of Cordero et al. [3] [18] who reported a negative association of catalase, CoQ_10_ with the disease symptom from FM patients as compared to normal. They correlated oxidative stress as measured by CoQ_10_ with headache in FM. According to them, the levels of an antioxidative enzyme such as SOD and catalase were decreased in the plasma of patients with FM and oxidative stress was higher caused by reactive oxygen species (ROS). A study by Bagis et al. [19] showed similar results on 85 women patients with primary FM and 80 healthy control women. They found lower levels of SOD and elevated levels of MDA (malondialdehyde) in the patients. Singh et al. [20] carried out a study of SOD, catalase and glutathione level on mice to clarify the severity of closely linked disorder the chronic fatigue syndrome and found lower levels of these variables. Eisinger et al. [12] also reported it decreased antioxidative parameters and increased oxidative stress in FMS patient. They studied in their study MDA as an indicator of lipid peroxidation, protein carbonyls and NO (nitrosothiols) and antioxidants including erythrocytes glyceraldehydes phosphate dehydrogenase (GAPDH), glutathione and SOD. They found significantly increased protein carbonyl and decreased NO values in the plasma and unchanged MDA values in the serum of FMS patients compared to healthy controls whereas the results of our study showed elevated levels of LPO and NO in the patients of FMS than in controls.

Our results of NO are consistent with those, reported by Bradley and co-workers [21], who found increase serum nitrite level in FMS patients compared to controls. Larson et al. [22] also explained the possible role of NO in FMS pain modulation. However, Ozgocemen et al. [23] found lower nitrite level in FM patient compared to control whereas Alasehirli et al. [24] and Sendur et al. [25] found no relationship between serum NO level and FM development but found a significant positive correlation between serum NO level and VAS pain in FM patient in Turkish population.

It is known that most FM patients present with a central sensitization phenomenon which consists of the exacerbation of neuronal responsiveness, particularly to noxious stimuli [26]. This phenomenon happens after repeated and frequent noxious stimuli, which in turn lead to a rearrangement of the plasticity of the central nervous system [27] [28]. Experimental studies have shown that NO is also an important neurotransmitter involved in the spinal pain pathway and that the sensitization of those pathways can occur or be related to nitric oxide synthase (NOS) activation with the subsequent rise of NO [29] [30]. NO plays an important part in vascular relaxation, blood flow regulation, and vasodilation induced through stress and in pain modulation [31], features that are known manifestations of FM. In our study, there was a positive association of NO and LPO with FIQR and negative correlation with the antioxidative defence system, that suggests the role of NO and LPO in the severity of FMS such as pain. It is proposed that lipid peroxidation is involved in the etiology of pain either by induction of central and peripheral hyperalgesia [32]. Although the mechanism of involvement of oxidative stress in pain modulation is not well known, it is believed that oxidative damage causes a local reduction of nociceptors and thereby reduce the pain threshold. According to available literature, NO may be involved in the initiation of LPO [33] by many putative ways, NO a donor of S-nitroso-N-acetyl penicillamine (SNAP) in U937 cells can inhibit glutathione peroxidase that leads to increased levels of lipid peroxidation [33]. Another way is the reaction of NO with molecular oxygen generates a radical-radical combination of product nitrogen dioxide (•NO_2_), which can initiate lipid peroxidation [8].

The elevated levels of NO and LPO and reduced level of GPx and negative association with antioxidative parameters in FMS patients and the positive linear association of NO and LPO with FIQR make us think that these factors together influence the symptoms and severity of FMS such as pain and fatigue.

## Conclusion

Elevated NO levels and oxidative stress causing factors such as LPO and reduced antioxidative enzyme activity, collectively may be responsible for the etiopathogenesis, symptoms and severity of FMS. Complete knowledge of the biochemical and genetic events occurring at the cellular level to influence nitric oxide increase level and oxidative damage is required to guide future research. However, it is difficult to determine whether the oxidative stress and NO are the cause or effect of FMS.

*Acknowledgement*. Financial support for this work was provided by the Indian Council of Medical Research, New Delhi, India (grant no. 5/4-ortho/2010-NCD-1)

Ethical Approval No. from the institutional ethical committee: Ref. Code: XL VI ECM/A-P9.

### Conflict of interest statement

The authors stated that they have no conflicts of interest regarding the publication of this article.
